# Sensitivity, specificity, inclusivity and exclusivity of the updated Aptima Combo 2 assay, which provides detection coverage of the new diagnostic-escape *Chlamydia trachomatis* variants

**DOI:** 10.1186/s12879-020-05148-7

**Published:** 2020-06-16

**Authors:** Magnus Unemo, Marit Hansen, Ronza Hadad, Mirja Puolakkainen, Henrik Westh, Kaisu Rantakokko-Jalava, Carina Thilesen, Michelle J. Cole, Iryna Boiko, Pham T. Lan, Daniel Golparian, Shin Ito, Martin Sundqvist

**Affiliations:** 1 World Health Organization Collaborating Centre for Gonorrhoea and Other Sexually Transmitted Infections (STIs), National Reference Laboratory for STIs, Department of Laboratory Medicine, Clinical Microbiology, Faculty of Medicine and Health, Örebro University, SE-701 85 Örebro, Sweden; 2 Department of Virology and Immunology, University of Helsinki and Helsinki University Hospital, HUSLAB, Helsinki, Finland; 3 Department of Clinical Medicine, Faculty of Medical Sciences, University of Copenhagen, Copenhagen, Denmark; 4 Department of Clinical Microbiology, Hvidovre University Hospital, Hvidovre, Denmark; 5 Department of Clinical Microbiology, Turku University Hospital, Turku, Finland; 6 Department of Microbiology, Unilabs Laboratory Medicine, Skien, Norway; 7 National Infection Service, Public Health England, London, UK; 8 Department of Functional and Laboratory Diagnostics, I. Horbachevsky Ternopil National Medical University, Ternopil, Ukraine; 9 Hanoi Medical University, National Hospital of Dermatology and Venereology, Hanoi, Vietnam; 10 iClinic, Sendai, Japan

**Keywords:** Finnish new variant, *Chlamydia trachomatis*, Aptima combo 2, 23S rRNA, Validation, Surveillance

## Abstract

**Background:**

Four new variants of *Chlamydia trachomatis* (nvCTs), detected in several countries, cause false-negative or equivocal results using the Aptima Combo 2 assay (AC2; Hologic). We evaluated the clinical sensitivity and specificity, as well as the analytical inclusivity and exclusivity of the updated AC2 for the detection of CT and *Neisseria gonorrhoeae* (NG) on the automated Panther system (Hologic).

**Methods:**

We examined 1004 clinical AC2 samples and 225 analytical samples spiked with phenotypically and/or genetically diverse NG and CT strains, and other potentially cross-reacting microbial species. The clinical AC2 samples included CT wild type (WT)-positive (n = 488), all four described AC2 diagnostic-escape nvCTs (n = 170), NG-positive (n = 214), and CT/NG-negative (n = 202) specimens.

**Results:**

All nvCT-positive samples (100%) and 486 (99.6%) of the CT WT-positive samples were positive in the updated AC2. All NG-positive, CT/NG-negative, *Trichomonas vaginalis* (TV)-positive, bacterial vaginosis-positive, and *Candida*-positive AC2 specimens gave correct results. The clinical sensitivity and specificity of the updated AC2 for CT detection was 99.7 and 100%, respectively, and for NG detection was 100% for both. Examining spiked samples, the analytical inclusivity and exclusivity were 100%, i.e., in clinically relevant concentrations of spiked microbe.

**Conclusions:**

The updated AC2, including two CT targets and one NG target, showed a high sensitivity, specificity, inclusivity and exclusivity for the detection of CT WT, nvCTs, and NG. The updated AC2 on the fully automated Panther system offers a simple, rapid, high-throughput, sensitive, and specific diagnosis of CT and NG, which can easily be combined with detection of *Mycoplasma genitalium* and TV*.*

## Background

Curable sexually transmitted infections (STIs) remain major global public health concerns [1]. *Chlamydia trachomatis* (CT) infection is the most common bacterial STI with 127 million estimated cases among adults each year globally [1]. In the European Union/European Economic Area (EU/EEA) and in many other international settings, the incidence of CT infections has increased during the past decade [1, 2]. The widespread use of highly sensitive and specific nucleic acid amplification tests (NAATs), particularly in high-income countries, has substantially contributed to the increased detection and incidence. Undetected and untreated CT infections can result in serious complications and sequelae, including infertility. CT infections are frequently asymptomatic and accordingly not detected if appropriate laboratory diagnostics is not performed [3].

Validated and quality-assured CT NAATs are recommended for highly sensitive and specific diagnosis of CT infections. Compared to other CT diagnostic methods, NAATs have highly superior sensitivities with maintained high specificities, use non-invasive specimens, are rapid, and provide opportunities for automation [3]. Initially, CT was considered to be genomically highly conserved. However, in 2006 the Swedish new variant of CT (nvCT) was described [4]. This Swedish nvCT has a deletion in the cryptic plasmid resulting in false negative results using the Roche and Abbott NAATs available at that time [5, 6]. Subsequently, whole genome sequencing of CT provided evidence of relatively substantial recombination and a mutational frequency level similar to the one in many other bacterial species [7, 8]. This showed that CT evolves more than previously predicted and diagnostic-escape CT mutants can emerge.

The sensitivity and specificity of the US FDA-approved Aptima Combo 2 assay (AC2; Hologic Inc., San Diego, CA, USA), based on target capture (TC) and transcription-mediated amplification (TMA) chemistries, for detection of CT (target: 23S rRNA) and *Neisseria gonorrhoeae* (NG; target: 16S rRNA) have proven excellent in many studies [9–17]. However, in early 2019, the Finnish nvCT (FI-nvCT) was identified [18, 19]. This variant has a single nucleotide polymorphism (SNP) in the CT 23S rRNA gene, i.e. C1515T (*Escherichia coli* numbering), resulting in escaped detection by the acridinium ester CT detection probe used in AC2 [18–24]. The FI-nvCT was initially estimated to represent 6–10% of the CT positive samples in Finland [18, 19]. The FI-nvCT has subsequently been reported in Sweden [20], Norway [21], and Denmark [24]. Three additional AC2 diagnostic-escape nvCTs have been reported in single specimens in Norway [21], the United Kingdom [25], and Japan (the present study), and one of these three nvCTs was found widely spread in Denmark [24]. These three AC2 diagnostic-escape nvCTs, as the FI-nvCT, have a SNP in the AC2 CT probe detection sequence of 23S rRNA, i.e. 23S rRNA C1514T, G1523A [21, 24, 25], or C1522T (the present study). The Aptima *C. trachomatis* NAAT (ACT; Hologic Inc.), which targets CT 16S rRNA, detects all of these diagnostic-escape nvCTs. Reflex testing of AC2 samples with relative light unit (RLU) values of 15–99 (mostly “high negative” or equivocal results) using ACT was implemented in May–June 2019 in many European countries [22, 26]. However, this ACT reflex testing substantially increases the work load and associated costs in these laboratories, and a more sustainable solution is imperative. Accordingly, an updated version of AC2, which was designed to detect also all these and other diagnostic-escape nvCTs, has now been developed by Hologic Inc. (San Diego, USA). This updated AC2 assay includes one additional CT detection probe targeting a second region of the CT 23S rRNA [27].

The aim of the present study was to evaluate the clinical sensitivity and specificity, as well as the analytical inclusivity and exclusivity of the updated AC2 (provided as research-use-only [RUO] material) for detection of wild-type (WT) CT, nvCTs, and NG on the automated Panther system (Hologic).

## Methods

The evaluation panel consisted of 1004 clinical, routinely collected samples (from 2015 to 2020) previously diagnosed using AC2 and/or ACT, in accordance with the instructions from the manufacturer (Hologic), and 225 samples spiked with geographically and temporally (from 1971 to 2016) diverse isolates of NG, CT, FI-nvCT, and *T. vaginalis* (TV), as well as non-NG *Neisseria*, *Moraxella*, non-TV *Trichomonas*, or non-CT *Chlamydia*.

### Clinical NAAT samples

The AC2 clinical samples consisted of CT WT-positive specimens (n = 488; 84 of these were also positive for NG); diagnostic-escape nvCTs (FI-nvCT [n = 114], nvCT-G1523A [n = 46; one positive also for NG], nvCT-C1522T [n = 6], and nvCT-C1514T [n = 4]; all verified by 23S rRNA gene sequencing or the FI-nvCT TMA assay [23]); NG-positive specimens (n = 214; 84 of these were also positive for CT); CT*-* and NG-negative specimens (n = 202); and specimens positive for TV (n = 5), bacterial vaginosis (n = 5), and *Candida* species (n = 5). The nvCT-samples comprised mainly first-void urine or vaginal/cervical samples from Finland, Sweden, Norway, Denmark, the United Kingdom, and Japan. Prior to testing with the updated AC2 assay, all clinical AC2 samples were collected and stored (− 20–70 °C) in routine diagnostics (standard care), and no patient identification information was available in the present study. All clinical AC2 samples were collected in Aptima Urine Specimen Transport Tubes or Aptima Vaginal Swab Specimen Collection Kit (Hologic). Results from the initial routine testing using the original version of AC2 were used for comparison against the updated AC2 results.

### Analytical inclusivity and exclusivity panel

The panel was assembled to include phenotypically and/or genetically diverse strains. For CT inclusivity, all nine main genotypes, including L1-L3 causing *Lymphogranuloma venereum* (LGV), and variants such as the Swedish nvCT [4–6], were represented. Furthermore, 20 samples including in vitro transcript (5 fg) of the FI-nvCT 23S rRNA (Panel G; Hologic) were examined. The four non-CT *Chlamydia* species *C. muridarium, C. suis, C. psittaci*, and *C. pneumoniae* were initially tested in high concentration (~ 1.1 × 10^7^ genome equivalents (GEQs)/test) to challenge the exclusivity. For NG inclusivity, the geographically, temporally and genomically diverse 2016 WHO reference strains (n = 14) [28], which include prolyliminopeptidase (PIP), *porA* pseudogene, and *cppB* mutants that have previously escaped diagnostics using other NAATs, were examined. Due to the high level of genetic homogeneity between NG and non-NG *Neisseria* species and problems with cross-reactivity in several NG NAATs [13], a high number of different strains (n = 151) representing 14 diverse non-NG *Neisseria* and three *Moraxella* species were tested in high concentration (~ 2 × 10^7^ genome equivalents (GEQs)/test) to substantially challenge the exclusivity. Finally, *T. vaginalis* samples (n = 10), including the two ATCC strains 30,001 and 50,140, and 10 samples including four different non-*T. vaginalis Trichomonas* species were tested in a high concentration (~ 1.25 × 10^7^ GEQs/test). All the preparation and testing of spiked samples were performed as previously described [29]. If any cross-reactivity was identified, the cross-reacting sample was diluted to more clinically relevant concentrations, i.e. the sample was diluted to contain similar number of GEQs as CT and NG are present in within strongly CT and NG positive samples [30–32].

### NAAT testing

Like the original AC2 formulation, the updated AC2 assay is based on TC and TMA chemistries. Testing with the updated AC2 (provided as RUO material) was performed on the Panther system (Hologic), in strict accordance with the instructions from the manufacturer. All testing was performed blinded in regards to the results from other testing.

## Results

The results of all testing of AC2 clinical samples and spiked samples with the updated AC2 are summarised in Table 1.

**Table 1:** Clinical and analytical detection of the updated Aptima Combo 2 assay (AC2; detecting CT and NG), which provides detection coverage of all diagnostic-escape new variants of CT [18–25, the present study]

Clinical specimen	Number tested	Positive AC2		Positive updated AC2	
		CT	NG	CT	NG
CT wild type (C1515)-positive clinical AC2 specimens^a,b^	488	488	84	486	84
FI-nvCT (C1515T)-positive clinical AC2 specimens^b^	114	0	0	114	0
nvCT-G1523A-positive clinical AC2 specimens^a,b^	46	0	1	46	1
nvCT-C1522T-positive clinical AC2 specimens^b^	6	0	0	6	0
nvCT-C1514T-positive clinical AC2 specimens^b^	4	0	0	4	0
*N. gonorrhoeae*-positive, CT-negative AC2 specimens	129	0	129	0	129
*T. vaginalis*-positive AC2 specimens	5	0	0	0	0
Bacterial vaginosis-positive AC2 specimens	5	0	0	0	0
*Candida* spp.-positive AC2 specimens	5	0	0	0	0
*C. trachomatis* and *N. gonorrhoeae*-negative clinical AC2 specimens	202	0	0	0	0
**Microbial species in spiked specimens**					
*C. trachomatis* Ba, D, E, F, G, H, J, K, L2b	9	9	0	9	0
*C. trachomatis* SE-nvCT [4–6]	1	1	0	1	0
FI-nvCT 23S rRNA in vitro transcript	20	0	0	20	0
*C. suis* (ATCC VR-1474)^c^	2	0	0	0	0
*C. muridarum* (ATCC VR-123)^c^	2	0	0	0	0
*C. pneumoniae* (ATCC VR-2282, MBC011)	4	0	0	0	0
*C. psittaci* (MBC013)	2	0	0	0	0
*Neisseria gonorrhoeae* 2016 WHO reference strains [28]	14	0	14	0	14
*N. animalis*	1	0	0	0	0
*N. bergeri*	1	0	0	0	0
*N. cinerea*	9	0	0	0	0
*N. elongata*^c^	3	0	0	0	0
*N. flava*	1	0	0	0	0
*N. flavescens*	32	0	0	0	0
*N. lactamica*	12	0	0	0	0
*N. macacae*	17	0	0	0	0
*N. mucosa*	17	0	0	0	0
*N. oralis*	1	0	0	0	0
*N. perflava*	22	0	0	0	0
*N. sicca*	9	0	0	0	0
*N. subflava*	6	0	0	0	0
*N. meningitidis*^d^	17	0	0	0	0
*Moraxella catarrhalis*	1	0	0	0	0
*M. nonliquefaciens*	1	0	0	0	0
*M. osloensis*	1	0	0	0	0
*Trichomonas vaginalis* (ATCC 30001, ATCC 50140)	10	0	0	0	0
*T. aotus* (ATCC 50649)	2	0	0	0	0
*T. gallinae* (ATCC 30002, 30,230)	4	0	0	0	0
*T. stableri* (ATCC PRA-412)	2	0	0	0	0
*T. tenax* (ATCC 30207)	2	0	0	0	0

AC2, Aptima Combo 2; CT, *Chlamydia trachomatis*; NG, *Neisseria gonorrhoeae*; FI-nvCT, Finnish new variant of CT; SE-nvCT, Swedish new variant of CT; ATCC, American Type Culture Collection; WHO, World Health Organization; RLU, relative light unit

^a^84 samples and one sample, respectively, were also confirmed positive for NG

^b^Confirmed by sequencing of the 23S rRNA gene or the FI-nvCT TMA assay [23]

^c^Positive in very high concentration, but negative in clinically more relevant concentrations

^d^Representing major meningococcal clones spreading worldwide, including serogroups A, B, C, E, W, X, Y and Z

### Clinical NAAT samples

All 170 nvCT-positive samples (FI-nvCT [n = 114], nvCT-G1523A [n = 46], nvCT-C1522T [n = 6], and nvCT-C1514T [n = 4]) and 486 (99.6%) of the 488 CT WT-positive samples were positive in the updated AC2. The two CT WT-positive samples that were negative in the updated AC2 showed RLU values of 14 and 8 in the updated AC2, while they were positive in the original AC2 (RLU values: 837 and 1215). All NG-positive (n = 214), CT/NG-negative (n = 202), *T. vaginalis*-positive (n = 5), bacterial vaginosis-positive (n = 5), and *Candida*-positive (n = 5) AC2 specimens gave correct results with the updated AC2 (Table 1). The overall clinical sensitivity and specificity of the updated AC2 for CT detection was 99.7% (CI 95%: 98.9–100%) and 100% (CI 95%: 98.9–100%), respectively. The overall clinical sensitivity and specificity for NG detection was 100% (CI 95%: 98.3–100%) and 100% (CI 95%: 99.5–100%), respectively.

### Analytical inclusivity and exclusivity panel

The inclusivity and exclusivity panel included samples spiked with the following: nine CT strains of different genotypes (n = 9) and the Swedish nvCT (n = 1); in vitro transcript of the FI-nvCT 23S rRNA (n = 20); four non-CT *Chlamydia* species (n = 10); all 2016 WHO NG reference strains (n = 14); 151 strains of non-NG *Neisseria* species (n = 14) and *Moraxella* species (n = 3); *T. vaginalis* samples, including the two ATCC strains 30,001 and 50,140 (n = 10); and five reference strains of four non-*T. vaginalis Trichomonas* species (n = 10). In clinically relevant concentrations, all samples gave correct results using the updated AC2 (Table 1).

Notably, in high concentrations (~ 2 × 10^7^ GEQs/test), two *N. elongata* strains were repeatedly yielding equivocal NG results with the updated AC2 (RLU values: 86–114 and 71–118, respectively). In clinically more relevant concentrations (~ 2 × 10^5^ GEQs/test), both strains were negative. Furthermore, in high concentrations (~ 1.1 × 10^7^ GEQs/test) *C. suis* (ATCC VR-1474) and *C. muridarum* (ATCC VR-123) tested CT positive with the updated AC2 (RLU values: 951 and 1159, respectively). However, in clinically more relevant concentrations (~ 1.1 × 10^5^ GEQs/test) both were negative (Table 1) and none of these animal pathogens have to our knowledge been found in human.

## Discussion

The present study is the first clinical and analytical validation of the updated AC2 NAAT (Hologic), which includes one NG target and two CT targets [27] and most recently was granted US FDA approval (https://www.accessdata.fda.gov/scripts/cdrh/cfdocs/cfPMN/pmn.cfm?ID=K200866). Since the initial report of the FI-nvCT in late-May 2019 in Finland [18, 19] and subsequent reports of FI-nvCT and additional diagnostic-escape nvCTs in Sweden [20], Norway [21], Denmark [24], and the United Kingdom [25], reflex testing with ACT of possible AC2 diagnostic-escape nvCT has been performed in all European diagnostic laboratories [22, 26]. However, Hologic has now developed an updated version of the AC2 assay [27], which was designed to detect also all of the new diagnostic-escape nvCTs [18–25, the present study] and includes one additional CT detection probe targeting a second region of the CT 23S rRNA. In the present study, we show that the updated AC2 assay has a maintained high clinical sensitivity and specificity, as well as an ideal analytical inclusivity and exclusivity, when examining samples spiked in clinically relevant concentrations of microbe, in the detection of WT CT and NG. Furthermore, the updated AC2 assay also effectively detected all of the described AC2 diagnostic-escape nvCTs [18–25, the present study], i.e. clinical AC2 specimens containing the FI-nvCT (n = 114), nvCT-G1523A (n = 46), nvCT-C1522T (n = 6), and nvCT-C1514T (n = 4) were all positive. Thus, the clinical sensitivity and specificity of the updated AC2 for CT detection was 99.7 and 100%, respectively. Despite that samples were preselected as positive with the original version of AC2, only two CT WT-positive samples were missed with the updated AC2 assay (not sufficient samples remaining for confirmation with ACT). It cannot be excluded that a preselection of samples using the updated AC2 assay would provide similar results when retested in the original AC2 assay. Both the clinical sensitivity and specificity of the updated AC2 assay for NG detection was 100%.

The recent emergence of AC2 diagnostic-escape nvCTs [18–25, the present study], spread of the Swedish nvCT escaping detection using the NAATs available from Roche and Abbott at that time [4–6, 33], and constantly evolving CT [7, 8], illustrate the necessity of dual target diagnostic CT NAATs and surveillance of the stability of NAAT targets in national and international settings. Furthermore, laboratories should be included in appropriate external quality assessments to detect diagnostic-escape nvCTs and it is imperative to adequately survey and analyse incidence, any unexplained shifts in positivity rates, and/or annual collections of samples diagnosed as negative/equivocal using NAATs with different target(s).

## Conclusions

Widespread implementation of validated, accurate and quality-assured CT dual target NAATs for diagnosis of CT is crucial internationally. The updated AC2 assay, now including two CT targets and one NG target [27], showed a high clinical sensitivity and specificity, as well as an ideal inclusivity and exclusivity in the detection of WT CT, all described nvCTs [4–6,18–25, the present study], and NG. Nevertheless, international and national surveillance programmes capturing NAAT diagnostic-escape variants for CT as well as other infectious agents are imperative.

## Data Availability

The datasets used and/or analysed during the current study are available from the corresponding author on reasonable request.

## Abbreviations

AC2: Aptima Combo 2
ACT: Aptima *Chlamydia trachomatis*
CT: *C. trachomatis*
EU/EEA: European Union/European Economic Area
fg: femtogram
FI-nvCT: Finnish new variant of *C. trachomatis*
GEQ: Genome equivalent
LGV: Lymphogranuloma venereum
NAAT: Nucleic acid amplification test
NG: *Neisseria gonorrhoeae*
nvCT: New variant of *C. trachomatis*
PIP: Prolyliminopeptidase
RUO: Research use only
SNP: Single nucleotide polymorphism
STI: Sexually transmitted infection
TC: Target capture
TMA: Transcription-mediated amplification
TV: *Trichomonas vaginalis*
WHO: World Health Organization
WT: Wild type
